# Agreement Between Standing Eight-Point Multifrequency Bioelectrical Impedance Analysis and Dual-Energy X-Ray Absorptiometry for Body Composition Assessment in Apparently Healthy Greek Adults

**DOI:** 10.3390/healthcare14121807

**Published:** 2026-06-22

**Authors:** Dimitrios Balampanos, Theodoros Stampoulis, Alexandra Avloniti, Anastasia Gkachtsou, Dimitrios Pantazis, Maria Protopapa, Nikolaos-Orestis Retzepis, Christos Kokkotis, Danai Kelaraki, Nikolaos Zaras, Dimitrios Ioannou, Stelios Kyriazidis, Maria Michalopoulou, Athanasios Chatzinikolaou

**Affiliations:** 1 Department of Physical Education and Sport Science, School of Physical Education, Sport Science and Occupational Therapy, Democritus University of Thrace, 69100 Komotini, Greece; dimibala10@phyed.duth.gr (D.B.); tstampou@ot.duth.gr (T.S.); alavloni@phyed.duth.gr (A.A.); anasgkac1@phyed.duth.gr (A.G.); dpantazi@phyed.duth.gr (D.P.); mprotopa@phyed.duth.gr (M.P.); nretzepi@phyed.duth.gr (N.-O.R.); ckokkoti@ot.duth.gr (C.K.); dkelarak@phyed.duth.gr (D.K.); nzaras@phyed.duth.gr (N.Z.); medthrace@otenet.gr (S.K.); michal@phyed.duth.gr (M.M.); 2 424 General Military Teaching Hospital, Nea Efkarpia, 56429 Thessaloniki, Greece; dimioa@gmail.com

**Keywords:** body fat mass, fat-free mass, BMI, validation

## Abstract

**Background/Objectives:** Multifrequency bioelectrical impedance analysis (MF-BIA) is increasingly used for practical body composition assessment when dual-energy X-ray absorptiometry (DXA) is unavailable or impractical. However, MF-BIA estimates are device-, population-, and outcome-specific, and therefore require validation against reference methods under standardized conditions. This study examined the agreement, concordance, and systematic bias between a standing 8-point MF-BIA device and DXA-derived body composition estimates in apparently healthy Greek adults. **Methods:** A total of 1250 adults aged 18 to 80 years completed same-day DXA and MF-BIA (Charder MA801) assessments. Fat mass (FM), fat-free mass (FFM), body fat percentage (BF%), and appendicular skeletal muscle mass estimate (ASM) were compared between methods. Analyses were performed by sex and BMI category. Pearson correlations described association, whereas Bland–Altman analysis, Lin’s concordance correlation coefficient (CCC), mean absolute error (MAE), root mean square error (RMSE), and proportional bias testing evaluated agreement and error magnitude. **Results:** MF-BIA showed strong associations with DXA-derived outcomes, but systematic bias was observed. When BMI categories were considered collectively, MF-BIA underestimated BF% by 3.59 percentage points in men and 4.25 percentage points in women, underestimated FM by 2.89 kg and 2.58 kg, and overestimated FFM by 3.09 kg and 3.29 kg, respectively. CCC was highest for FM (men: 0.913; women: 0.949) and lower for FFM and ASM in women (0.642 and 0.714, respectively). Proportional bias was observed for BF%, FM, and ASM in both sexes, and for FFM in women. **Conclusions:** The MA801 showed strong associations and outcome-specific concordance with DXA, but systematic bias and individual-level error limit interchangeability. Under standardized conditions, MF-BIA may support group-level or repeated same-device assessments but not precise individual-level assessment, clinical classification, or monitoring of small longitudinal changes.

## 1. Introduction

Body composition assessment is important for identifying health-related differences in fat mass, lean soft tissue, hydration status, and skeletal integrity, which are associated with conditions such as malnutrition, sarcopenia, osteoporosis, frailty, obesity, and cardiometabolic disease [1,2,3,4,5]. Beyond total adiposity, regional fat distribution is also clinically relevant, as central fat accumulation is more strongly associated with adverse metabolic and cardiovascular outcomes than peripheral fat deposition [1]. However, the usefulness of any body composition method depends not only on feasibility but also on whether its estimates are sufficiently accurate for the intended application. A method may be appropriate for field-based screening, epidemiological assessment, or group-level monitoring yet still be insufficiently precise for individual diagnosis, classification, or monitoring of small longitudinal changes.

Dual-energy X-ray absorptiometry (DXA) is widely used as a reference method for body composition assessment because it provides estimates of fat mass, lean soft tissue, and bone mineral content with low radiation exposure and relatively short scan time [6,7]. Nevertheless, its routine use is limited by cost, restricted accessibility, the need for specialized equipment and trained personnel, and practical constraints such as device weight limits [6,8]. Simpler anthropometric methods, including skinfold thickness, body mass index (BMI), and body circumferences, are inexpensive and widely available, but they are limited by operator dependence or by their inability to distinguish fat mass from lean mass [9,10,11]. These limitations have increased interest in bioelectrical impedance analysis (BIA), a practical, non-invasive, radiation-free, and relatively low-cost method for estimating body composition.

BIA does not directly measure fat mass, muscle mass, or lean tissue. Instead, it estimates body composition from electrical resistance and reactance through prediction equations influenced by device configuration, electrode placement, current frequency, body geometry, hydration assumptions, and population characteristics [12,13,14,15,16]. Early single-frequency BIA systems, typically operating at 50 kHz, had limited ability to characterize fluid distribution across body compartments, whereas multifrequency BIA (MF-BIA) applies current at several frequencies and may improve the estimation of body-water distribution [14]. Despite this theoretical advantage, the accuracy of MF-BIA remains dependent on the embedded prediction model and on the population in which the device is used. This is especially important because commercial MF-BIA devices often rely on proprietary equations that are not fully transparent, making device-specific validation necessary before results are generalized across populations or applications.

Recent evidence reinforces the need for device- and population-specific evaluation of BIA-derived body composition estimates. In a large UK Biobank analysis, Feng et al. compared BIA- and DXA-derived fat mass and fat-free mass using Lin’s concordance correlation coefficient, Bland–Altman analysis, Passing–Bablok analysis, and multivariable regression [17]. Although high population-level concordance was observed, BIA underestimated fat mass and overestimated fat-free mass, while between-method differences were associated with body composition and anthropometric characteristics, including BMI and waist circumference [17]. Recent MF-BIA studies have also shown that measurement repeatability and agreement with reference methods are influenced by standardization factors such as hydration status, recent physical activity, prandial state, and time of day [18,19]. Furthermore, contemporary agreement-focused studies increasingly combine Bland–Altman analysis with concordance metrics and calibration models, emphasizing that strong statistical association does not necessarily indicate individual-level interchangeability [20,21].

The distinction between correlation, concordance, and agreement is critical in method-comparison studies. Pearson correlation coefficients indicate the extent to which two methods rank participants similarly, but they do not determine whether the absolute values generated by the two methods are sufficiently close to be used interchangeably [22]. Lin’s concordance correlation coefficient provides additional information by evaluating both precision and deviation from the line of identity, whereas Bland–Altman analysis quantifies mean bias and limits of agreement [22,23]. Therefore, agreement between MF-BIA and DXA should be evaluated using complementary statistical approaches that assess association, concordance, systematic bias, limits of agreement, and potential proportional bias, rather than relying solely on correlation coefficients. This is particularly relevant for MF-BIA because measurement error may vary according to sex, BMI category, adiposity, hydration status, and regional tissue distribution [17,21].

An additional consideration is that validation evidence from one MF-BIA system cannot be extrapolated uncritically to another. MF-BIA devices differ in electrode configuration, measurement posture, frequency range, segmentation approach, and proprietary prediction algorithms. Consequently, findings from other commercial systems, such as InBody or Tanita devices, should not be assumed to apply directly to the Charder MA801. Although standing 8-point MF-BIA devices may be useful in applied, epidemiological, and clinical settings because they are rapid and easy to administer, their practical value depends on their agreement and concordance with DXA-derived estimates under standardized conditions.

Therefore, the objective of the present study was to examine the agreement, concordance, and systematic bias between a standing 8-point MF-BIA device and DXA-derived body composition estimates in a large sample of apparently healthy Greek adults from a regional setting in Greece. This population-specific validation is relevant because MF-BIA performance may vary according to population characteristics, body size, adiposity distribution, hydration-related assumptions, and device-specific prediction algorithms. Specifically, we evaluated fat mass, fat-free mass, body fat percentage, and appendicular skeletal muscle mass estimate, with DXA-derived appendicular lean soft tissue mass used as the proxy reference for ASM. Analyses were stratified by sex and BMI category. By examining agreement, concordance, systematic bias, and sex- and BMI-related patterns, this study aimed to clarify the practical utility and limitations of this MF-BIA device for body composition assessment under standardized laboratory conditions.

## 2. Materials and Methods

### 2.1. Participants

A total of 1250 apparently healthy adults aged 18 to 80 years participated in this cross-sectional method-comparison study. Participants were recruited through the Department of Physical Education and Sport Science, Democritus University of Thrace, Komotini, Greece. Recruitment was based on non-probability convenience sampling; therefore, the sample should be interpreted as a large volunteer cohort of apparently healthy adults rather than as a population-representative cohort. Eligible participants were adults who could stand independently and complete both dual-energy X-ray absorptiometry (DXA) and multifrequency bioelectrical impedance analysis (MF-BIA) assessments during the same laboratory visit. Participants were considered apparently healthy based on self-reported health status and ability to complete the assessment protocol. Individuals were not eligible if they had conditions or characteristics that could interfere with DXA or MF-BIA assessment, including pregnancy, implanted electronic medical devices, limb amputation, inability to stand unaided, acute illness, visible edema, or known conditions associated with marked fluid imbalance. Seven individuals who self-reported known cardiometabolic disease or other health conditions incompatible with the assessment protocol were excluded. However, cardiometabolic status was not systematically verified through clinical examination, medical record review, medication review, or biochemical screening. All participants underwent DXA and MF-BIA assessment on the same day. DXA was performed first, followed by MF-BIA after a standardized standing period.

Before participation, all individuals were informed about the study’s purpose and procedures and provided written informed consent. The study was conducted in accordance with the Declaration of Helsinki and was approved by the Ethics Committee of Democritus University of Thrace (Protocol No. A.Π.Δ.Π.Θ./Ε.Δ.Ε./62808/581; approved on 25 July 2019). Physical activity level and training status were not formally assessed using a validated questionnaire. Accordingly, the sample should be interpreted as an apparently healthy adult cohort, but not as a cohort characterized according to sedentary, recreationally active, athletic, or trained status.

### 2.2. Study Design and Pre-Assessment Procedures

Participants attended the laboratory once for anthropometric and body composition assessment. To standardize pre-assessment conditions, participants were instructed to complete a 12 h fast, arrive with an empty bladder, refrain from caffeine and alcohol consumption for at least 12 h, and avoid strenuous physical activity for at least 48 h before testing. Female participants were not assessed from 2 days before to 2 days after the onset of menstruation to minimize the potential influence of menstrual-cycle-related fluid retention on body composition measurements. Testing was performed under standardized laboratory conditions. When feasible, assessments were scheduled during the morning hours to reduce the potential influence of diurnal variation on hydration status and impedance-derived estimates.

Before assessment, compliance with pre-test instructions was verified by participant self-report. Hydration status was standardized indirectly by fasting, bladder emptying, and avoiding alcohol, caffeine, and strenuous exercise before testing. However, hydration status was not directly assessed using urine specific gravity, urine osmolality, or biochemical markers. DXA assessment was performed first. After the DXA scan, participants remained standing for 10 min before the MF-BIA assessment to reduce the potential influence of posture-related fluid shifts following the supine DXA measurement.

#### 2.2.1. Body Height

Body height was measured using a portable stadiometer (HM202P, Charder Electronic Co., Ltd., Taichung City, Taiwan). Participants were assessed barefoot and without headwear, standing upright with the head positioned in the Frankfort horizontal plane and the arms relaxed at the sides. Height was recorded to the nearest 0.1 cm. Two measurements were obtained, and their mean value was retained for analysis.

#### 2.2.2. MF-BIA Assessment and Body Mass Measurement

Whole-body and segmental body composition were assessed using a multifrequency bioelectrical impedance analyzer with an 8-point standing hand-to-foot electrode system (Charder MA801, Charder Electronic Co., Ltd., Taichung City, Taiwan). The device uses eight electrodes and operates at five frequencies: 5, 20, 50, 100, and 250 kHz. According to the manufacturer’s documentation, the MA801 is a professional body composition analyzer that conducts multifrequency bioelectrical impedance analysis and provides whole-body and segmental body composition estimates. The device manual also states that BIA results are calculated from impedance values using population-based statistical models; however, the embedded prediction equations are proprietary and were not available to the investigators. Therefore, the present study evaluated the agreement of the device outputs with DXA-derived estimates but could not independently examine the mathematical structure of the device-specific prediction models.

MF-BIA assessment was performed according to the manufacturer’s instructions and standardized laboratory procedures previously described by Balampanos et al. [24,25]. Before measurement, participants removed shoes, socks, and metal objects and emptied their bladders. Participants stood barefoot on the electrode platform, ensuring direct contact between the soles of the feet and the foot electrodes, and held the hand electrodes with both hands. During the measurement, participants remained upright and motionless, with the arms positioned slightly away from the trunk to avoid contact between the arms and torso. The measurement was performed only after stable electrode contact had been established.

Body mass obtained from the same device was used to calculate body mass index (BMI = kg/m^2^). MF-BIA-derived variables included fat mass (FM), fat-free mass (FFM), body fat percentage (BF%), and appendicular skeletal muscle mass estimate (ASM). Each MF-BIA assessment was performed twice during the same visit under standardized conditions. Within-session repeatability was evaluated using the two repeated measurements, and the mean value was retained for the agreement analyses with DXA.

#### 2.2.3. DXA

Whole-body and regional body composition were assessed using dual-energy X-ray absorptiometry (Lunar iDXA, GE Healthcare, Madison, WI, USA), according to the manufacturer’s instructions and standardized laboratory procedures previously described by Balampanos et al. [24]. Before scanning, participants removed all metal objects to avoid interference with the measurement. Participants were positioned supine on the scanning table, with the body aligned centrally within the scanning area, the arms placed alongside the trunk, and the lower limbs positioned according to the manufacturer’s whole-body scan protocol. Participants were instructed to remain still throughout the scan.

The DXA system was maintained and serviced in accordance with the manufacturer’s procedures by the authorized service provider. Phantom quality control was conducted on each testing day before participant assessments using the manufacturer-provided calibration phantom (GE Healthcare, Madison, WI, USA). Quality-control scans were accepted only when calibration values were within the manufacturer-specified tolerance limits. All scans were performed by trained laboratory personnel using a standardized positioning and acquisition protocol. Scan acquisition and region-of-interest analysis were performed using enCORE software, version 14.10.022 (GE Healthcare, Madison, WI, USA), including the whole-body and segmental body composition analysis module. Automated region-of-interest segmentation was visually inspected and corrected when necessary according to the manufacturer’s analysis procedures.

DXA-derived variables included fat mass (FM), lean soft tissue mass, total mass, and body fat percentage (BF%). DXA-derived appendicular lean soft tissue mass was calculated from segmental analysis as the sum of lean soft tissue mass from both arms and legs and was used as the proxy reference for ASM.

### 2.3. Statistical Analysis

Body composition variables obtained from MF-BIA and DXA were analyzed using an agreement-based method-comparison framework. Analyses were performed separately by sex and BMI category. BMI categories were defined as normal weight (18.5 ≤ BMI < 25 kg/m^2^), overweight (25 ≤ BMI < 30 kg/m^2^), and obesity (BMI ≥ 30 kg/m^2^). Exploratory age-stratified sensitivity analyses were also performed using three broad age categories: 18–39 years, 40–59 years, and ≥60 years. These analyses were conducted descriptively for BF%, FFM, and FM to examine whether between-method bias patterns were broadly consistent across age ranges. Broad age categories were used to avoid excessive fragmentation of the sample and unstable estimates from combined sex × BMI × age strata. Descriptive statistics are presented as mean ± standard deviation and range.

Within-session repeatability of duplicate MF-BIA measurements was assessed for FM and FFM in all participants. Repeatability was quantified using intraclass correlation coefficients [ICC(3,1)], Cronbach’s alpha, Lin’s concordance correlation coefficient (CCC), Pearson’s correlation coefficient, coefficient of variation (CV), mean absolute error (MAE), root mean square error (RMSE), mean bias, and 95% limits of agreement. The mean of the two MF-BIA measurements was retained for all agreement analyses with DXA.

Pearson correlation coefficients with 95% confidence intervals were calculated to describe the strength of association between MF-BIA- and DXA-derived BF%, FFM, FM, and ASM. Pearson correlations were interpreted only as indices of association and not as evidence of agreement or interchangeability between methods.

The standard error of the estimate (SEE) was calculated from the linear regression of DXA-derived values on MF-BIA-derived values for each outcome and subgroup. SEE was used as a supplementary regression-based index of residual error around the fitted regression line and was reported in the same units as the corresponding body composition variable. SEE was interpreted as complementary to, and not as a replacement for, the primary agreement metrics.

Agreement between MF-BIA and DXA was evaluated using Bland–Altman analysis. Bias was calculated as MF-BIA minus DXA; therefore, positive values indicated overestimation by MF-BIA, whereas negative values indicated underestimation by MF-BIA. The 95% limits of agreement were calculated as the mean bias ± 1.96 times the standard deviation of the between-method differences.

Lin’s concordance correlation coefficient (CCC), with 95% confidence intervals, was calculated for BF%, FFM, FM, and ASM to evaluate concordance between MF-BIA and DXA by accounting for both precision and deviation from the line of identity. Lin’s CCC was interpreted together with Bland–Altman bias and limits of agreement, rather than as a replacement for agreement analysis.

The magnitude of error was further quantified using mean absolute error (MAE) and root mean square error (RMSE). MAE was calculated as the mean absolute between-method difference, and RMSE was calculated as the square root of the mean squared between-method difference. Proportional bias was assessed by regressing the between-method difference on the pairwise mean. A statistically significant regression slope was interpreted as evidence of magnitude-dependent measurement error. These analyses were performed for BF%, FFM, FM, and ASM.

Formal heteroscedasticity was assessed using the Breusch–Pagan test applied to the residuals of the proportional-bias model, in which the between-method difference was regressed on the mean of the two methods. This analysis examined whether residual variance changed across the measurement range. Breusch–Pagan testing was performed for BF%, FFM, and FM, consistent with the outcomes included in the supplementary heteroscedasticity analyses.

Mean differences between MF-BIA- and DXA-derived variables were examined using paired-samples *t*-tests. Given the large sample size, *p*-values from paired comparisons were interpreted with caution and were not considered sufficient evidence of practical agreement. Standardized paired mean differences were calculated as Cohen’s dz, defined as the mean paired difference divided by the standard deviation of the paired differences. Cohen’s dz values were interpreted descriptively as standardized indices of paired between-method differences, not as evidence of agreement or interchangeability.

ASM was analyzed as a continuous body composition outcome. DXA-derived appendicular lean soft tissue mass was used as the proxy reference for ASM. Clinical cross-classification analyses around obesity- or sarcopenia-related thresholds were not performed because diagnostic classification was not a prespecified aim of the study.

The primary interpretation of method comparability was based on mean bias, limits of agreement, and Lin’s CCC for all outcomes, with MAE, RMSE, and proportional bias additionally considered for BF%, FFM, FM, and ASM. Statistical significance was set at *p* < 0.05. Analyses were conducted using IBM SPSS Statistics for Windows, Version 26.0 (IBM Corp., Armonk, NY, USA). Supplementary analyses included standardized paired mean differences (Supplementary Table S1), exploratory age-stratified agreement analyses (Supplementary Table S2), and additional proportional-bias slopes and Breusch–Pagan heteroscedasticity analyses for BF%, FFM, and FM (Supplementary Table S3).

## 3. Results

### 3.1. Demographic and Anthropometric Characteristics

Anthropometric characteristics of the total sample are shown in Table 1. The sample included 688 men and 562 women, with women showing a slightly higher mean age and lower mean body mass than men.

### 3.2. Within-Session Repeatability

Within-session repeatability was assessed using two consecutive MF-BIA measurements performed during the same visit in all 1250 participants. Repeatability was excellent for both FM and FFM (Table 2). For FM, Pearson’s r was 0.9988, ICC(3,1) was 0.9988, Cronbach’s alpha was 0.9994, Lin’s CCC was 0.9988, and CV was 2.21%. For FFM, Pearson’s r was 0.9997, ICC(3,1) was 0.9997, Cronbach’s alpha was 0.9998, Lin’s CCC was 0.9997, and CV was 0.61%. Mean bias between duplicate measurements was small for both variables, indicating very high within-session consistency under the standardized MF-BIA protocol.

### 3.3. Association, Concordance, and Overall Agreement Between MF-BIA and DXA

**Figure 1:**
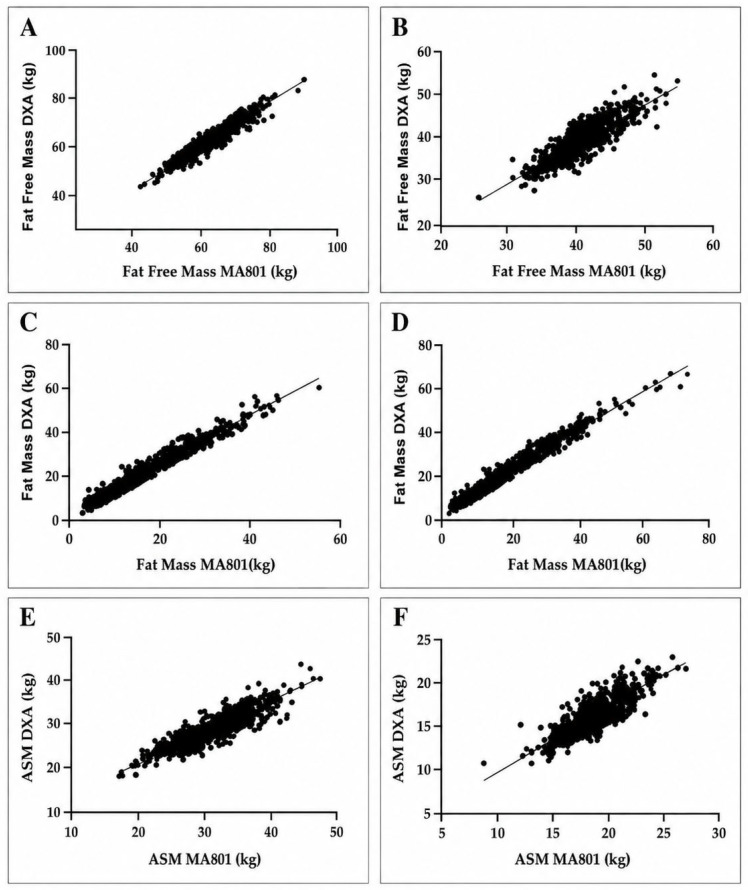
Associations between MF-BIA- and DXA-derived fat-free mass, fat mass, and appendicular skeletal muscle mass estimated by sex. Panels (**A**,**B**) show fat-free mass (FFM) in men and women, respectively. Panels (**C**,**D**) show fat mass (FM) in men and women, respectively. Panels (**E**,**F**) show appendicular skeletal muscle mass estimate (ASM) in men and women, respectively. The x-axis represents MF-BIA-derived values from the MA801, and the y-axis represents DXA-derived values. Each dot represents one participant, and the solid line represents the fitted linear regression line.

Pearson correlation coefficients showed strong positive associations between MF-BIA- and DXA-derived body composition variables in both sexes (Table 3). Scatterplots for FFM, FM, and ASM are presented in Figure 1. In the overall sex-specific analysis, correlations ranged from r = 0.863 for ASM to r = 0.967 for FM in men and from r = 0.822 for ASM to r = 0.981 for FM in women. BMI-stratified correlation coefficients are presented in Table 4, Table 5 and Table 6. Across BMI categories, correlations ranged from r = 0.919 to 0.932 for FFM in men and from r = 0.880 to 0.899 in women, from r = 0.848 to 0.927 for FM in men and from r = 0.888 to 0.954 in women, from r = 0.802 to 0.908 for BF% in men and from r = 0.766 to 0.891 in women, and from r = 0.767 to 0.894 for ASM in men and from r = 0.804 to 0.888 in women. However, Lin’s CCC values showed that concordance varied by outcome and sex. Concordance was highest for FM in both men and women (CCC = 0.913 and 0.949, respectively), whereas FFM and ASM showed lower concordance in women (CCC = 0.642 and 0.714, respectively).

**Figure 2:**
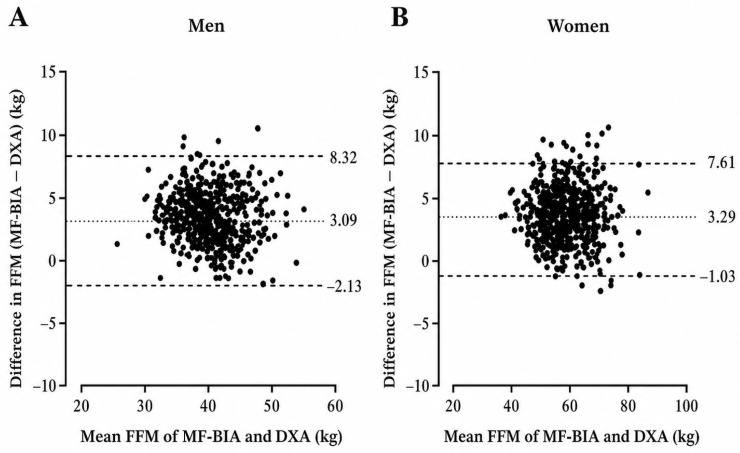
Bland–Altman plots showing agreement between MF-BIA- and DXA-derived fat-free mass (FFM) in men and women. Panel (**A**) presents men, and Panel (**B**) presents women. Each dot represents one participant. The central line represents the mean bias, calculated as MF-BIA minus DXA, and the dashed lines represent the 95% limits of agreement. Positive values indicate overestimation by MF-BIA, whereas negative values indicate underestimation by MF-BIA.

**Figure 3:**
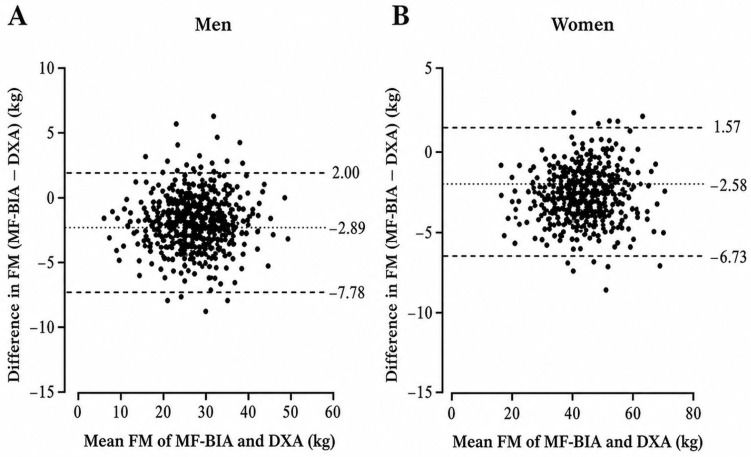
Bland–Altman plots showing agreement between MF-BIA- and DXA-derived fat mass (FM) in men and women. Panel (**A**) presents men, and Panel (**B**) presents women. Each dot represents one participant. The central line represents the mean bias, calculated as MF-BIA minus DXA, and the dashed lines represent the 95% limits of agreement. Positive values indicate overestimation by MF-BIA, whereas negative values indicate underestimation by MF-BIA.

Bland–Altman analysis showed systematic between-method bias for all primary body composition outcomes (Table 3). Bland–Altman plots for FFM and FM are presented in Figure 2 and Figure 3. Additional Bland–Altman plots are provided for BF% in Supplementary Figure S1 and for ASM in Supplementary Figure S2 to support visual inspection of agreement patterns for these outcomes. Bias was calculated as MF-BIA minus DXA; therefore, positive values indicate MF-BIA overestimation and negative values indicate underestimation. In men, MF-BIA underestimated BF% by 3.59 percentage points and FM by 2.89 kg, whereas FFM was overestimated by 3.09 kg. ASM showed a small underestimation of 0.35 kg. In women, MF-BIA underestimated BF% by 4.25 percentage points and FM by 2.58 kg, whereas FFM and ASM were overestimated by 3.29 kg and 1.23 kg, respectively.

Error magnitude was quantified using MAE and RMSE for BF%, FFM, FM, and ASM (Table 3). In men, MAE values were 3.92 percentage points for BF%, 3.39 kg for FFM, 3.18 kg for FM, and 1.55 kg for ASM, while RMSE values were 4.67 percentage points, 4.09 kg, 3.82 kg, and 1.99 kg, respectively. In women, MAE values were 4.58 percentage points for BF%, 3.43 kg for FFM, 2.82 kg for FM, and 1.54 kg for ASM, while RMSE values were 5.30 percentage points, 3.96 kg, 3.34 kg, and 1.83 kg, respectively. Proportional bias was observed for BF%, FM, and ASM in both sexes, and for FFM in women, indicating that disagreement between MF-BIA and DXA varied across the measurement range for these outcomes.

Additional proportional-bias slopes and Breusch–Pagan heteroscedasticity analyses for BF%, FFM, and FM are presented in Supplementary Table S3. Breusch–Pagan testing indicated heteroscedasticity for FFM and FM in both sexes, whereas heteroscedasticity was not detected for BF% in either sex.

Exploratory age-stratified sensitivity analyses for BF%, FFM, and FM are provided in Supplementary Table S2. Because age groups were not evenly distributed across sex and BMI categories, these analyses were interpreted descriptively and were not used to replace the primary sex- and BMI-stratified agreement analyses.

### 3.4. BMI-Stratified Agreement

BMI-stratified analyses showed that the direction of bias was generally consistent across BMI categories, although the magnitude of disagreement varied by sex and BMI group (Table 4, Table 5 and Table 6). For FFM, MF-BIA overestimated DXA-derived values across all BMI categories. In men, FFM bias was 2.75 kg in the normal-weight group, 3.51 kg in the overweight group, and 3.28 kg in the obesity group. In women, FFM bias was 2.71 kg, 4.47 kg, and 4.19 kg, respectively.

For FM, MF-BIA underestimated DXA-derived values across all BMI categories. In men, FM bias was −2.71 kg in the normal-weight group, −3.23 kg in the overweight group, and −2.63 kg in the obesity group. In women, FM bias was −2.09 kg, −3.65 kg, and −3.22 kg, respectively.

For BF%, MF-BIA underestimated DXA-derived values across all BMI categories. In men, BF% bias was −3.67 percentage points in the normal-weight group, −3.76 in the overweight group, and −2.69 in the obesity group. In women, BF% bias was −3.80, −5.54, and −4.39 percentage points, respectively.

ASM showed a different pattern by sex. In men, MF-BIA underestimated ASM in the normal-weight and overweight groups by −0.73 kg and −0.21 kg, respectively, but overestimated ASM in the obesity group by 0.90 kg. In women, MF-BIA overestimated ASM across all BMI categories, with biases of 0.91 kg in the normal-weight group, 1.82 kg in the overweight group, and 1.83 kg in the obesity group.

### 3.5. Paired Method Comparisons

Paired comparisons between MF-BIA- and DXA-derived values are presented in Table 7 and Table 8. In the overall sample and across BMI categories, MF-BIA-derived FFM was significantly higher than DXA-derived FFM in both sexes. MF-BIA-derived FM and BF% were significantly lower than DXA-derived values in most sex- and BMI-specific comparisons. For ASM, significant differences were observed primarily in women, where MF-BIA-derived ASM was higher than DXA-derived ASM across BMI categories. In men, ASM differences were smaller and varied by BMI category.

Standardized paired mean differences are presented in Supplementary Table S1. The direction of Cohen’s dz values was consistent with the Bland–Altman mean bias estimates, with positive values indicating overestimation by MF-BIA relative to DXA and negative values indicating underestimation. These values were interpreted descriptively as standardized indices of paired between-method differences, not as evidence of agreement or interchangeability.

Given the large sample size, *p*-values were interpreted together with bias, limits of agreement, Lin’s CCC, MAE, RMSE, and proportional-bias results rather than as standalone evidence of method agreement.

## 4. Discussion

The present study examined the agreement, concordance, and systematic bias between a standing 8-point MF-BIA device and DXA-derived body composition estimates in apparently healthy Greek adults. The central finding was that strong associations between the Charder MA801 and DXA-derived estimates coexisted with systematic, outcome-specific bias and non-negligible individual-level error. MF-BIA underestimated BF% and FM and overestimated FFM in both sexes, while ASM showed a sex-specific pattern of bias. This pattern is practically relevant because the MA801 tended to provide a more favorable estimation of body composition than DXA, characterized by lower adiposity and higher FFM estimates. However, this finding should be interpreted with caution, as these differences reflect method-related bias rather than true physiological differences. Therefore, although the MA801 preserved between-individual ranking reasonably well, it did not reproduce DXA-derived values closely enough to be considered interchangeable with DXA for precise individual-level interpretation.

This distinction addresses a key methodological issue in body composition validation research. Pearson correlation coefficients indicate whether individuals with higher DXA-derived values also tend to have higher MF-BIA-derived values, but they do not determine whether the absolute values produced by the two methods are close enough to be used interchangeably [22]. In the present study, this limitation was clear. Although Pearson correlations were strong, Lin’s concordance correlation coefficient (CCC) provided a more conservative interpretation because it accounts for both precision and deviation from the line of identity [23]. In men, CCC values were 0.857 for BF%, 0.863 for FFM, 0.913 for FM, and 0.858 for ASM. In women, CCC values were 0.852 for BF%, 0.642 for FFM, 0.949 for FM, and 0.714 for ASM. The lower CCC values for FFM and ASM in women show that a strong association did not necessarily translate into close concordance for these variables. Therefore, the present findings should be interpreted primarily through agreement and error-based metrics rather than through Pearson correlations alone.

The practical meaning of the observed bias should be interpreted in relation to the intended use of the measurement. Body composition estimates are used in clinical, research, and applied settings to interpret adiposity, lean soft tissue, and muscle-related status, but the consequences of measurement error depend on whether the method is used for group-level description, individual classification, or longitudinal monitoring [1,6,10,26]. In the present study, MF-BIA underestimated BF% by approximately 3.6 percentage points in men and 4.3 percentage points in women, while FM was underestimated by approximately 2.9 kg and 2.6 kg, respectively. At the same time, FFM was overestimated by approximately 3.1 kg in men and 3.3 kg in women. This error pattern shifts interpretation toward a more favorable body composition profile, characterized by lower adiposity and higher FFM estimates than those obtained with DXA. Such bias may be less consequential when the objective is broad group-level estimation under standardized conditions, but it is not negligible for individual interpretation. In obesity-related assessment, underestimation of adiposity may affect interpretation because BMI does not distinguish fat mass from fat-free mass and may misclassify body composition profiles [9]. Similarly, overestimation of FFM and ASM may be relevant in nutritional, functional, athletic, and sarcopenia-related interpretation, where FFM or appendicular lean soft tissue estimates are often considered alongside health, function, or performance-related outcomes [10,27]. This error pattern does not, by itself, establish diagnostic misclassification, but it indicates that MF-BIA-derived values should be interpreted cautiously when individual values are close to clinically or practically meaningful thresholds. In such cases, the direction of bias may shift the interpretation of adiposity, FFM, or appendicular muscle-related status compared with DXA-derived estimates. Mechanistically, the observed bias may partly reflect assumptions embedded in the MA801 prediction model regarding body-water distribution, tissue conductivity, and the relationship between impedance values and body composition compartments [12,13,14,15,16]. Sex-related differences in fat distribution, limb-to-trunk tissue distribution, and relative fat-free mass may influence current pathways and the conversion of impedance-derived values into compartment estimates, while higher adiposity may alter the relationship between resistance, reactance, extracellular water distribution, and predicted fat-free mass [14,17,21,28]. However, these mechanisms were not directly tested because hydration compartments, raw impedance-model assumptions, and proprietary prediction equations were not available for independent examination. This limitation is particularly relevant when considering the clinical interpretation of MF-BIA-derived estimates. The present study evaluated agreement and concordance between MF-BIA- and DXA-derived estimates, but it did not examine diagnostic classification performance. Therefore, the findings should not be interpreted as evidence that the MA801 can accurately classify individuals according to obesity- or sarcopenia-related thresholds.

The error-magnitude metrics further clarify the practical consequences of the observed disagreement. For BF%, RMSE values were 4.67 percentage points in men and 5.30 percentage points in women, while MAE values were 3.92 and 4.58 percentage points, respectively. For FFM, RMSE values were 4.09 kg in men and 3.96 kg in women, with MAE values of 3.39 and 3.43 kg, respectively. For FM, RMSE values were 3.82 kg in men and 3.34 kg in women, with MAE values of 3.18 and 2.82 kg, respectively. These values indicate that disagreement was not limited to statistically significant mean differences but extended to individual-level error of a magnitude that may affect interpretation. In practical terms, MAE and RMSE define the scale of uncertainty that should be considered when interpreting individual MF-BIA-derived values. This is particularly important for longitudinal monitoring because an observed change should be interpreted with caution when its magnitude is comparable to the method’s expected measurement error [29,30]. Therefore, small changes in BF%, FM, or FFM should not be interpreted as true biological changes unless they clearly exceed the expected error range of the measurement approach.

The present findings place the Charder MA801 within the broader group of MF-BIA systems that preserve a strong association with DXA-derived values but still show systematic, outcome-specific disagreement. The direction of bias observed here, namely lower adiposity estimates and higher FFM estimates compared with DXA, is consistent with large-scale and BMI-focused BIA-DXA evidence. Feng et al. reported that BIA underestimated FM and overestimated FFM in the UK Biobank despite strong population-level concordance [17], while Achamrah et al. and Pateyjohns et al. reported similar patterns of adiposity underestimation and FFM overestimation in overweight- and obesity-focused samples [30,31]. Taken together, these findings suggest that multifrequency, segmental BIA systems may produce broadly comparable bias directions relative to DXA, particularly in terms of adiposity underestimation and FFM overestimation. However, this consistency should not be interpreted as evidence of device interchangeability, because MF-BIA systems differ in frequency architecture, electrode configuration, posture, segmentation model, and proprietary prediction algorithms. In this context, the MA801 appears to show an intermediate agreement profile within the comparative literature: its bias was not as large as that reported in some higher-BMI or clinical samples, but the observed disagreement remained too large to support direct individual-level substitution for DXA.

This position becomes clearer when the MA801 is considered alongside other commercial MF-BIA systems. Lahav et al. directly compared SECA mBCA 515 and InBody 770 with DXA across BMI categories and showed that the two devices did not behave identically: SECA produced BF% estimates closer to DXA, whereas InBody 770 underestimated BF%, with BMI category influencing the magnitude of deviation [32]. This comparison is especially relevant because it shows that MF-BIA performance cannot be inferred from the technology label alone. The number and range of frequencies may influence the measurement profile, but frequency architecture does not determine accuracy independently. Devices using multiple frequencies may still differ because of electrode configuration, standing or supine posture, current pathway, segmental modeling, body-water assumptions, population-specific calibration, and proprietary equations [14,28].

Relative to studies reporting favorable agreement for some direct segmental MF-BIA systems, the MA801 showed a more outcome-specific agreement pattern. Ling et al. reported strong agreement between direct segmental MF-BIA and DXA for total and segmental body composition in middle-aged adults, and Buch et al. reported high agreement between InBody 770 and DXA for muscle-related outcomes in older adults with type 2 diabetes [33,34]. These findings support the broader view that modern direct segmental MF-BIA devices, particularly systems using multiple frequencies and multi-electrode configurations, can provide useful body composition estimates when applied under standardized conditions. However, the present findings show that this favorable performance cannot be assumed to extend uniformly across devices, populations, and outcomes. For the MA801, the most favorable agreement profile was observed for FM, whereas FFM and ASM, particularly in women, showed weaker concordance despite moderate-to-strong correlations. This pattern suggests that the MA801 may be more suitable for broad group-level adiposity or fat-mass assessment than for precise individual-level interpretation of FFM or appendicular muscle-related status.

The comparison with studies reporting weaker interchangeability for muscle-related estimates further supports this caution. Lee et al. showed that BIA and DXA were not necessarily interchangeable for whole-body and appendicular muscle-related estimates [35]. The present findings align with that concern, especially for ASM and FFM in women. This is important because commercial MF-BIA devices do not directly measure FFM or ASM; they estimate these compartments through internal prediction models. For the MA801, the embedded equations were proprietary and unavailable for independent examination. Therefore, the present study can evaluate how closely MA801 outputs agreed with DXA-derived estimates under standardized laboratory conditions, but it cannot isolate whether the observed bias was driven primarily by the five-frequency architecture, standing 8-point hand-to-foot electrode design, segmental assumptions, hydration modeling, or the internal prediction equation.

Taken together, the comparative literature supports a cautious but practically useful interpretation of the MA801. The device preserved between-individual ranking and provided meaningful group-level information, especially for FM, while showing smaller disagreement than that reported in some higher-BMI or clinical samples. At the same time, its performance was outcome-specific, with weaker concordance for FFM and ASM in women. These findings suggest that the MA801 can be useful for standardized group-level assessment and repeated same-device monitoring, particularly when DXA is unavailable or impractical. However, because systematic bias and proportional error were present, the device should not be considered a direct substitute for DXA when precise individual classification or small longitudinal changes are the main objective.

The BMI-stratified findings align with studies showing that BIA-DXA agreement may vary across BMI and body composition profiles. Achamrah et al. reported BMI-related variation in agreement between BIA and DXA, and Shafer et al. showed that segmental MF-BIA performance differs across BMI ranges [30,36]. Feng et al. also found that BIA-DXA differences were related to anthropometric and body composition characteristics in a large cohort [17]. In the present study, the direction of bias was generally stable across BMI categories, but the magnitude of disagreement varied by sex and BMI group. This matters because total-sample agreement may obscure subgroup-specific error patterns. However, because the present analyses were stratified descriptively rather than modeled inferentially, sex and BMI should not be interpreted as independently tested predictors of bias. Future studies should formally model determinants of MF-BIA error, including sex, BMI, waist circumference, age, hydration markers, and physical activity or training status [14,16,28].

The proportional-bias findings strengthen this interpretation. Proportional bias was observed for BF%, FM, and ASM in both sexes, and for FFM in women, indicating that disagreement between MF-BIA and DXA changed across the measurement range for several outcomes. Thus, a single average bias value does not describe device performance equally well across individuals with lower or higher body composition values. For FFM, proportional bias was not observed in men but was present in women, consistent with the weaker CCC observed for FFM in women. For ASM, proportional bias in both sexes further supports the interpretation that MF-BIA-derived ASM estimates should not be treated as directly interchangeable with DXA-derived appendicular lean soft tissue mass. This pattern suggests that error was not purely random, but partly dependent on body composition level and sex-specific body composition profiles. From a practical perspective, this limits the usefulness of simple correction factors and supports device- and population-specific interpretation.

Recent MF-BIA studies also reinforce the importance of standardized testing conditions and formal reliability assessment. Potter et al. examined MF-BIA performance under real-world conditions where factors such as hydration, prandial status, recent exercise, and time of day were not tightly controlled, while Looney et al. highlighted the importance of reliability and biological variability when interpreting MF-BIA-derived body composition estimates [18,19]. These studies support the broader point that MF-BIA performance is not determined only by the analyzer, but also by testing context and biological variability. In the present study, pre-assessment conditions were standardized, but hydration was not directly verified and physical activity or training status was not formally assessed. Therefore, the observed bias should be interpreted as the agreement between MA801-derived estimates and DXA-derived estimates under the present protocol, not as evidence of broader device reliability across testing conditions.

The ASM findings warrant particular caution because appendicular muscle-related estimates are often used in clinically meaningful contexts, including sarcopenia screening. Previous studies have shown that agreement between BIA and DXA for appendicular muscle-related estimates is device- and population-specific [34,35,37]. In the present study, MF-BIA overestimated ASM in women, and concordance for ASM in women was weaker than for FM. Practically, this upward bias may make some women appear to have a more favorable appendicular muscle profile than would be suggested by DXA-derived appendicular lean soft tissue mass, particularly when values are close to sarcopenia-related decision thresholds. Because DXA-derived appendicular lean soft tissue mass was used as the proxy reference for ASM, these findings should not be interpreted as direct validation against true skeletal muscle mass. These findings do not establish diagnostic misclassification, because cross-classification against sarcopenia-related cut-offs was not performed. However, they indicate that borderline cases should be interpreted with particular caution, since even modest systematic error may shift an individual above or below a clinically relevant threshold [26]. Accordingly, MA801-derived ASM should not be treated as interchangeable with DXA-derived appendicular lean soft tissue mass for screening or classification until diagnostic validation around sarcopenia-related cut-offs is available.

The findings clarify the appropriate practical use of the device. The strongest use of the Charder MA801 is not as a DXA replacement but as a practical estimation tool for standardized, repeated, same-device assessment. From an applied perspective, the MA801 may be useful in settings where DXA is unavailable, impractical, or unnecessary for the specific decision context, including large-sample epidemiological assessments, primary-care or community screening contexts, obesity-related monitoring, sports medicine assessments, and repeated same-device tracking under standardized conditions. However, MA801 outputs should not be interpreted as DXA-equivalent values. In obesity-related contexts, underestimation of BF% and FM may shift interpretation toward a more favorable adiposity profile, whereas in sarcopenia-related contexts, overestimation of FFM and ASM may be relevant when individuals are close to clinically meaningful thresholds. Therefore, MF-BIA may support practical body composition estimation, but it should be applied with awareness of outcome-specific bias and expected measurement error.

The present study has several strengths. These include the large sample size, inclusion of both sexes, broad adult age range, same-day assessment using both MF-BIA and DXA, standardized pre-assessment procedures, and sex- and BMI-stratified analyses. In addition, the use of complementary agreement and error metrics strengthened the interpretation of the findings by distinguishing association, concordance, mean bias, individual-level error, and proportional bias. This is important because a device may rank individuals similarly to DXA while still producing values that are not close enough for direct substitution. The findings also align with the main error pattern reported by Feng et al. in the UK Biobank cohort, as BIA underestimated adiposity-related outcomes and overestimated FFM-related outcomes compared with DXA [17]. This supports the relevance of the present results beyond a single Greek cohort while still indicating that the magnitude of disagreement should be interpreted in relation to the specific device, population, and outcome.

Several limitations should be considered when interpreting these findings. One limitation concerns the sampling strategy and participant characterization. The sample consisted of apparently healthy Greek adults recruited through non-probability volunteer-based sampling; therefore, the findings should not be generalized uncritically to other ethnic groups, clinical populations, individuals with altered hydration status, or populations with extreme body composition profiles without further validation. The sample was not characterized in detail regarding physical activity level, training status, metabolic health, medication use, menopausal status, or cardiometabolic risk profile. Although participants who self-reported known cardiometabolic disease or other health conditions incompatible with the assessment protocol were not included, cardiometabolic status was not systematically verified through clinical examination, medical record review, medication review, or biochemical screening. This is relevant because these factors may influence hydration status, tissue characteristics, body-water distribution, and impedance-derived estimates. Although exploratory age-stratified sensitivity analyses are provided in Supplementary Table S2, the study was not specifically powered for detailed age-stratified or age-modeled validation; therefore, future studies should examine age as a potential determinant of MF-BIA error using appropriately powered designs. In women, menopausal and broader hormonal status were not formally assessed. Although assessments in menstruating women were scheduled outside the perimenstrual period when applicable, the absence of menopausal-status characterization limits the interpretation of sex-specific agreement patterns, particularly given the broad age range of the female sample. Future validation studies should therefore record menopausal status and hormonal status when examining MF-BIA accuracy in adult female populations.

Another limitation relates to hydration status. Hydration status was standardized indirectly through pre-assessment instructions, fasting, bladder emptying, and avoidance of caffeine, alcohol, and strenuous exercise, but it was not directly verified using urine specific gravity, urine osmolality, or biochemical markers. A further limitation concerns the reference method itself. Although DXA is widely used and accepted as a reference method for body composition assessment, it has methodological limitations and should not be interpreted as a criterion method in the strictest sense. DXA-derived estimates may be influenced by hydration status, device and software differences, participant positioning, segmentation procedures, and assumptions regarding tissue composition [37,38]. Therefore, the present findings should be interpreted as agreement with DXA-derived estimates rather than as validation against an absolute reference standard. Multicompartment models, particularly four-compartment approaches, provide a more robust reference framework for body composition validation because they reduce reliance on assumptions related to fat-free mass composition and hydration.

In addition, the proprietary prediction equations embedded in the MA801 were not available to the investigators, limiting interpretation of the internal sources of prediction error. Another methodological limitation is that two consecutive MF-BIA measurements were performed in all participants and averaged for the agreement analyses with DXA. Within-session repeatability was formally evaluated for FM and FFM, showing excellent short-term consistency for both variables. However, repeatability indices were not calculated for BF% and ASM; therefore, the repeatability of these MF-BIA-derived outputs should be interpreted with greater caution. Moreover, duplicate DXA scans were not performed in the present protocol, and participant-level DXA test–retest repeatability could not be calculated from this dataset. Nevertheless, the precision of GE Lunar iDXA for total and regional body composition assessment has been previously established in repeated-scan studies with repositioning, including excellent precision for lean soft tissue mass, fat mass, and percentage body fat [39,40]. In the present study, the quality of DXA measurements was supported by authorized maintenance, daily phantom quality control, trained personnel, and standardized acquisition and analysis procedures. Finally, underweight individuals and participants at the upper extreme of the obesity range were underrepresented, and cross-classification analyses around obesity- or sarcopenia-related thresholds were not performed because diagnostic classification was not a prespecified aim of the study.

In summary, the Charder MA801 showed strong associations and outcome-specific concordance with DXA-derived body composition estimates but also demonstrated systematic bias and individual-level error. The device tended to underestimate BF% and FM and overestimate FFM, while ASM showed sex-specific bias. These findings support the use of MF-BIA as a practical estimation method under standardized conditions, particularly for group-level or repeated same-device assessment in similar apparently healthy adult populations. However, MF-BIA should not be considered interchangeable with DXA for precise individual-level assessment, clinical classification, or monitoring of small longitudinal changes.

## 5. Conclusions

In this large sample of apparently healthy Greek adults, the Charder MA801 showed a strong association and outcome-specific concordance with DXA-derived body composition estimates but also demonstrated systematic bias. MF-BIA tended to underestimate BF% and FM and overestimate FFM, while ASM showed a sex-specific bias pattern. These findings suggest that, under standardized conditions, the MA801 may support practical body composition assessment, particularly for group-level interpretation or repeated same-device monitoring in similar populations. However, the observed agreement patterns do not support interchangeability with DXA for precise individual-level assessment, clinical classification, or interpretation of small longitudinal changes. Future studies should examine whether these biases affect classification around prespecified obesity- and sarcopenia-related thresholds and should model factors that may influence MF-BIA error, including sex, BMI, hydration status, and physical activity or training status.

## Figures and Tables

**Table 1:** Participants’ characteristics.

Sex	Age (Years)	Height (cm)	Body Mass (kg)	BMI (kg/m^2^)
Male (*n* = 688)	31.2 ± 13.2 (18.0–72.7)	179.4 ± 7.2 (159.0–201.0)	82.6 ± 13.4 (56.4–129.3)	25.6 ± 3.7 (18.9–41.6)
Female (*n* = 562)	37.6 ± 17.3 (18.0–79.6)	164.5 ± 6.5 (134.9–181.0)	66.2 ± 11.9 (41.5–116.6)	24.6 ± 4.8 (18.5–47.4)

BMI: Body mass index. Data are presented as mean ± standard deviation (minimum–maximum).

**Table 2:** Within-session repeatability of duplicate MF-BIA measurements.

Variable	n	Pearson’s r	ICC(3,1)	Cronbach’s α	Lin’s CCC	CV (%)	RMSE	MAE	Bias	95% LoA
FM (kg)	1250	0.9988	0.9988	0.9994	0.9988	2.21	0.3222	0.2356	0.0471	−0.5812 to 0.6754
FFM (kg)	1250	0.9997	0.9997	0.9998	0.9997	0.61	0.3198	0.2322	−0.0437	−0.6683 to 0.5809

Note: MF-BIA: multifrequency bioelectrical impedance analysis; FM: fat mass; FFM: fat-free mass; r: Pearson’s correlation coefficient; ICC: intraclass correlation coefficient; α: Cronbach’s alpha; CCC: concordance correlation coefficient; CV: coefficient of variation; RMSE: root mean square error; MAE: mean absolute error; LoA: limits of agreement. Repeatability was assessed from two consecutive MF-BIA measurements performed during the same visit. Bias was calculated as measurement 2 minus measurement 1.

**Table 3:** Overall association, concordance, agreement, and error magnitude between MF-BIA- and DXA-derived body composition estimates by sex.

Variable	Sex	n	Pearson’s r (95% CI)	Lin’s CCC (95% CI)	Bias (95% CI)	Bias (SD)	95% LoA	MAE	RMSE	Proportional Bias
BF%	Men	688	0.937 (0.927 to 0.946)	0.857 (0.841 to 0.872)	−3.59 (−3.81 to −3.37)	−3.59 (2.99)	−9.44 to 2.26	3.92 pp	4.67 pp	Present, *p* < 0.001
FFM (kg)	Men	688	0.935 (0.925 to 0.944)	0.863 (0.843 to 0.880)	3.09 (2.89 to 3.29)	3.09 (2.67)	−2.13 to 8.32	3.39 kg	4.09 kg	Not present, *p* = 0.923
FM (kg)	Men	688	0.967 (0.962 to 0.972)	0.913 (0.900 to 0.923)	−2.89 (−3.08 to −2.70)	−2.89 (2.50)	−7.78 to 2.00	3.18 kg	3.82 kg	Present, *p* < 0.001
ASM (kg)	Men	688	0.863 (0.843 to 0.881)	0.858 (0.837 to 0.877)	−0.35 (−0.50 to −0.20)	−0.35 (1.95)	−4.18 to 3.48	1.55 kg	1.99 kg	Present, *p* = 0.007
BF%	Women	562	0.946 (0.937 to 0.954)	0.852 (0.835 to 0.867)	−4.25 (−4.51 to −3.99)	−4.25 (3.17)	−10.47 to 1.97	4.58 pp	5.30 pp	Present, *p* < 0.001
FFM (kg)	Women	562	0.874 (0.853 to 0.892)	0.642 (0.521 to 0.726)	3.29 (3.11 to 3.47)	3.29 (2.20)	−1.03 to 7.61	3.43 kg	3.96 kg	Present, *p* = 0.019
FM (kg)	Women	562	0.981 (0.978 to 0.984)	0.949 (0.940 to 0.957)	−2.58 (−2.76 to −2.40)	−2.58 (2.12)	−6.73 to 1.57	2.82 kg	3.34 kg	Present, *p* < 0.001
ASM (kg)	Women	562	0.822 (0.792 to 0.846)	0.714 (0.680 to 0.745)	1.23 (1.12 to 1.34)	1.23 (1.36)	−1.44 to 3.90	1.54 kg	1.83 kg	Present, *p* = 0.001

Note: DXA: dual-energy X-ray absorptiometry; MF-BIA: multifrequency bioelectrical impedance analysis; BF%: body fat percentage; FFM: fat-free mass; FM: fat mass; ASM: appendicular skeletal muscle mass estimate; r: Pearson’s correlation coefficient; CI: confidence interval; CCC: concordance correlation coefficient; SD: standard deviation; LoA: limits of agreement; MAE: mean absolute error; RMSE: root mean square error; pp: percentage points. Bias was calculated as MF-BIA minus DXA. Positive values indicate overestimation by MF-BIA, and negative values indicate underestimation by MF-BIA. MAE, RMSE, and proportional-bias analyses were calculated for BF%, FFM, FM, and ASM.

**Table 4:** Association and agreement between MF-BIA and DXA in normal-weight participants. BMI category: 18.5 ≤ BMI < 25 kg/m^2^; men n = 353, women n = 363.

	Men (n = 353)				Women (n = 363)			
Variable	r (95% CI)	SEE	Bias (SD)	95% LoA	r (95% CI)	SEE	Bias (SD)	95% LoA
FFM (kg)	0.928 (0.912–0.941)	2.37	2.75 (2.45)	−2.06 to 7.56	0.880 (0.854–0.901)	2.02	2.71 (2.02)	−1.25 to 6.66
FM (kg)	0.848 (0.816–0.875)	2.28	−2.71 (2.28)	−7.19 to 1.77	0.936 (0.922–0.948)	1.85	−2.09 (1.93)	−5.88 to 1.70
BF%	0.814 (0.776–0.846)	3.05	−3.67 (3.06)	−9.66 to 2.33	0.891 (0.868–0.910)	3.18	−3.80 (3.28)	−10.22 to 2.63
ASM (kg)	0.894 (0.871–0.913)	1.47	−0.73 (1.51)	−3.69 to 2.22	0.804 (0.764–0.838)	1.29	0.91 (1.36)	−1.75 to 3.57

**Table 5:** Association and agreement between MF-BIA and DXA in overweight participants. BMI category: 25 ≤ BMI < 30 kg/m^2^; men n = 256, women n = 118.

	Men (n = 256)				Women (n = 118)			
Variable	r (95% CI)	SEE	Bias (SD)	95% LoA	r (95% CI)	SEE	Bias (SD)	95% LoA
FFM (kg)	0.932 (0.914–0.946)	2.80	3.51 (2.80)	−1.99 to 9.00	0.899 (0.858–0.929)	1.88	4.47 (1.99)	0.56 to 8.38
FM (kg)	0.927 (0.908–0.942)	2.50	−3.23 (2.61)	−8.33 to 1.88	0.888 (0.842–0.921)	2.01	−3.65 (2.02)	−7.60 to 0.30
BF%	0.908 (0.884–0.927)	2.88	−3.76 (2.97)	−9.58 to 2.06	0.787 (0.707–0.847)	2.64	−5.54 (2.72)	−10.87 to −0.20
ASM (kg)	0.863 (0.828–0.891)	2.10	−0.21 (2.11)	−4.34 to 3.91	0.886 (0.840–0.919)	1.01	1.82 (1.19)	−0.51 to 4.15

**Table 6:** Association and agreement between MF-BIA and DXA in participants with obesity. BMI category: BMI ≥ 30 kg/m^2^; men n = 79, women n = 81.

	Men (n = 79)				Women (n = 81)			
Variable	r (95% CI)	SEE	Bias (SD)	95% LoA	r (95% CI)	SEE	Bias (SD)	95% LoA
FFM (kg)	0.919 (0.876–0.948)	2.75	3.28 (2.90)	−2.40 to 8.97	0.886 (0.828–0.925)	2.30	4.19 (2.30)	−0.32 to 8.70
FM (kg)	0.894 (0.839–0.931)	2.81	−2.63 (2.89)	−8.29 to 3.03	0.954 (0.929–0.970)	2.19	−3.22 (2.29)	−7.71 to 1.28
BF%	0.802 (0.706–0.869)	2.40	−2.69 (2.53)	−7.65 to 2.26	0.766 (0.658–0.843)	2.64	−4.39 (2.75)	−9.77 to 1.00
ASM (kg)	0.767 (0.657–0.845)	2.47	0.90 (2.53)	−4.07 to 5.86	0.888 (0.831–0.927)	0.96	1.83 (1.11)	−0.35 to 4.01

Note: DXA: dual-energy X-ray absorptiometry; MF-BIA: multifrequency bioelectrical impedance analysis; BMI: body mass index; BF%: body fat percentage; FFM: fat-free mass; FM: fat mass; ASM: appendicular skeletal muscle mass estimate; r: Pearson’s correlation coefficient; CI: confidence interval; SEE: standard error of the estimate; SD: standard deviation; LoA: limits of agreement. Bias was calculated as MF-BIA minus DXA. Positive values indicate overestimation by MF-BIA, and negative values indicate underestimation by MF-BIA. Confidence intervals for Pearson’s r were calculated using Fisher’s z transformation.

**Table 7:** MF-BIA- and DXA-derived body composition values in men by BMI category.

Variable	Method	Overall (n = 688)	Normal Weight (n = 353)	Overweight (n = 256)	Obesity (n = 79)
FFM (kg)	MF-BIA	66.2 ± 7.4 (45.1–93.7)	63.8 ± 6.5 (45.1–85.1)	67.8 ± 7.2 (48.9–93.7)	71.7 ± 7.4 (55.7–92.4)
	DXA	63.1 ± 7.4 (42.1–89.4)	61.1 ± 6.4 (42.1–81.3)	64.3 ± 7.8 (44.6–89.4)	68.4 ± 7.0 (52.1–88.0)
	*p*-value	<0.001	<0.001	<0.001	0.005
FM (kg)	MF-BIA	16.4 ± 9.3 (2.1–55.1)	9.8 ± 3.6 (2.1–20.7)	19.8 ± 5.5 (7.6–32.7)	34.7 ± 6.3 (25.3–55.1)
	DXA	19.3 ± 9.8 (4.2–54.8)	12.5 ± 4.3 (4.2–25.1)	23.0 ± 6.7 (7.1–40.0)	37.3 ± 6.3 (25.6–54.8)
	*p*-value	<0.001	<0.001	<0.001	0.010
BF%	MF-BIA	18.9 ± 8.1 (3.0–44.8)	13.2 ± 4.4 (3.0–26.1)	22.5 ± 5.5 (9.1–34.0)	32.5 ± 4.0 (25.7–44.8)
	DXA	22.5 ± 8.6 (6.7–46.2)	16.9 ± 5.3 (6.7–31.1)	26.2 ± 6.9 (10.0–43.2)	35.2 ± 4.0 (25.2–46.2)
	*p*-value	<0.001	<0.001	<0.001	<0.001
ASM (kg)	MF-BIA	27.9 ± 3.6 (18.0–40.7)	26.7 ± 3.3 (18.0–38.0)	28.7 ± 3.5 (19.2–40.7)	30.6 ± 3.5 (22.4–39.5)
	DXA	28.2 ± 3.8 (18.0–43.3)	27.4 ± 3.3 (18.0–37.6)	28.9 ± 4.2 (18.2–43.3)	29.7 ± 3.9 (21.2–42.3)
	*p*-value	0.079	0.003	0.531	0.130

**Table 8:** MF-BIA- and DXA-derived body composition values in women by BMI category.

Variable	Method	Overall (n = 562)	Normal Weight (n = 363)	Overweight (n = 118)	Obesity (n = 81)
FFM (kg)	MF-BIA	44.0 ± 4.3 (28.1–58.4)	43.1 ± 3.8 (33.6–55.7)	44.4 ± 4.5 (28.1–55.2)	47.3 ± 4.3 (36.9–58.4)
	DXA	40.7 ± 4.5 (27.2–55.4)	40.4 ± 4.3 (29.8–53.4)	39.9 ± 4.3 (27.2–50.3)	43.1 ± 5.0 (31.7–55.4)
	*p*-value	<0.001	<0.001	<0.001	<0.001
FM (kg)	MF-BIA	22.2 ± 9.7 (6.0–67.0)	16.5 ± 4.4 (6.0–27.8)	27.7 ± 4.0 (17.6–39.9)	39.4 ± 7.7 (28.0–67.0)
	DXA	24.8 ± 10.5 (4.2–67.4)	18.6 ± 5.2 (4.2–33.3)	31.4 ± 4.4 (18.0–44.5)	42.6 ± 7.3 (31.5–67.4)
	*p*-value	<0.001	<0.001	<0.001	0.007
BF%	MF-BIA	32.3 ± 8.5 (13.8–57.5)	27.4 ± 5.5 (13.8–42.6)	38.4 ± 4.0 (26.5–47.3)	45.2 ± 3.9 (35.8–57.5)
	DXA	36.5 ± 9.6 (8.3–60.2)	31.2 ± 7.0 (8.3–47.7)	43.9 ± 4.3 (30.4–53.3)	49.6 ± 4.1 (38.4–60.2)
	*p*-value	<0.001	<0.001	<0.001	<0.001
ASM (kg)	MF-BIA	18.1 ± 2.3 (8.2–25.7)	17.8 ± 2.2 (11.5–24.6)	18.1 ± 2.6 (8.2–24.9)	19.2 ± 2.4 (13.6–25.7)
	DXA	16.8 ± 2.2 (11.1–23.4)	16.9 ± 2.2 (11.1–23.4)	16.3 ± 2.2 (11.2–22.0)	17.4 ± 2.1 (13.2–22.1)
	*p*-value	<0.001	<0.001	<0.001	<0.001

Note: Data are presented as mean ± standard deviation (minimum–maximum). DXA: dual-energy X-ray absorptiometry; MF-BIA: multifrequency bioelectrical impedance analysis; BMI: body mass index; BF%: body fat percentage; FFM: fat-free mass; FM: fat mass; ASM: appendicular skeletal muscle mass estimate. *p*-values refer to paired comparisons between MF-BIA- and DXA-derived values within each sex and BMI category.

## Data Availability

The data presented in this study are available on reasonable request from the corresponding author. The data are not publicly available due to privacy and ethical restrictions.

## Supplementary Materials

The following supporting information can be downloaded at: https://www.mdpi.com/article/10.3390/healthcare14121807/s1, Supplementary Table S1: Standardized paired mean differences between MF-BIA- and DXA-derived body composition estimates; Supplementary Figure S1: Bland–Altman plots comparing MF-BIA- and DXA-derived body fat percentage (BF%) in men and women; Supplementary Figure S2: Bland–Altman plots comparing MF-BIA- and DXA-derived appendicular skeletal muscle mass estimate (ASM) in men and women; Supplementary Table S2: Exploratory age-stratified agreement between MF-BIA- and DXA-derived body composition estimates; Supplementary Table S3: Proportional-bias and Breusch–Pagan heteroscedasticity analyses.
