# Supplementary material for: Agreement Between Standing Eight-Point Multifrequency Bioelectrical Impedance Analysis and Dual-Energy X-Ray Absorptiometry for Body Composition Assessment in Apparently Healthy Greek Adults
Source: Healthcare (Basel). 2026 Jun 22;14(12):1807. doi: 10.3390/healthcare14121807 (PMC13299440; doi:10.3390/healthcare14121807)
Supplement: Supplementary file 1 [file healthcare-14-01807-s001.zip › healthcare-4263164-Supplementary.pdf]

Supplementary Table S1. Standardized paired mean differences between MF-BIA- and DXA-derived body composition estimates

Variable	Sex	BMI category	n	Bias (SD)	Cohen's dz
BF%	Men	Overall	688	−3.59 (2.99)	−1.20
BF%	Men	Normal weight	353	−3.67 (3.06)	−1.20
BF%	Men	Overweight	256	−3.76 (2.97)	−1.27
BF%	Men	Obesity	79	−2.69 (2.53)	−1.06
BF%	Women	Overall	562	−4.25 (3.17)	−1.34
BF%	Women	Normal weight	363	−3.80 (3.28)	−1.16
BF%	Women	Overweight	118	−5.54 (2.72)	−2.04
BF%	Women	Obesity	81	−4.39 (2.75)	−1.60
FFM (kg)	Men	Overall	688	3.09 (2.67)	1.16
FFM (kg)	Men	Normal weight	353	2.75 (2.45)	1.12
FFM (kg)	Men	Overweight	256	3.51 (2.80)	1.25
FFM (kg)	Men	Obesity	79	3.28 (2.90)	1.13
FFM (kg)	Women	Overall	562	3.29 (2.20)	1.50
FFM (kg)	Women	Normal weight	363	2.71 (2.02)	1.34
FFM (kg)	Women	Overweight	118	4.47 (1.99)	2.25
FFM (kg)	Women	Obesity	81	4.19 (2.30)	1.82
FM (kg)	Men	Overall	688	−2.89 (2.50)	−1.16
FM (kg)	Men	Normal weight	353	−2.71 (2.28)	−1.19
FM (kg)	Men	Overweight	256	−3.23 (2.61)	−1.24
FM (kg)	Men	Obesity	79	−2.63 (2.89)	−0.91
FM (kg)	Women	Overall	562	−2.58 (2.12)	−1.22
FM (kg)	Women	Normal weight	363	−2.09 (1.93)	−1.08
FM (kg)	Women	Overweight	118	−3.65 (2.02)	−1.81

Variable	Sex	BMI category	n	Bias (SD)	Cohen's dz
FM (kg)	Women	Obesity	81	−3.22 (2.29)	−1.41
ASM (kg)	Men	Overall	688	−0.35 (1.95)	−0.18
ASM (kg)	Men	Normal weight	353	−0.73 (1.51)	−0.48
ASM (kg)	Men	Overweight	256	−0.21 (2.11)	−0.10
ASM (kg)	Men	Obesity	79	0.90 (2.53)	0.36
ASM (kg)	Women	Overall	562	1.23 (1.36)	0.90
ASM (kg)	Women	Normal weight	363	0.91 (1.36)	0.67
ASM (kg)	Women	Overweight	118	1.82 (1.19)	1.53
ASM (kg)	Women	Obesity	81	1.83 (1.11)	1.65

Note: BF%, body fat percentage; FFM, fat-free mass; FM, fat mass; ASM, appendicular skeletal muscle mass estimate; DXA, dual-energy X-ray absorptiometry; MF-BIA, multifrequency bioelectrical impedance analysis; BMI, body mass index; SD, standard deviation. Bias was calculated as MF-BIA minus DXA. Positive values indicate overestimation by MF-BIA, whereas negative values indicate underestimation by MF-BIA. Cohen's dz was calculated as the mean paired difference divided by the standard deviation of the paired differences and was interpreted only as a descriptive standardized index of paired between-method difference, not as evidence of agreement or interchangeability.

Supplementary Figure S1. Bland–Altman plots comparing MF-BIA- and DXA-derived body fat percentage (BF%) in men and women.

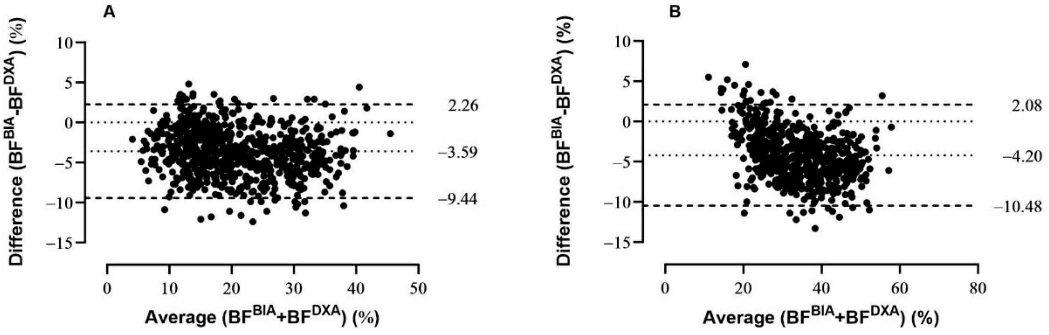

Panel A presents men, and Panel B presents women. The central dotted line represents the mean bias, calculated as MF-BIA minus DXA, and the dashed lines represent the 95% limits of agreement. Positive values indicate overestimation by MF-BIA, whereas negative values indicate underestimation by MF-BIA.

Supplementary Figure S2. Bland–Altman plots comparing MF-BIA- and DXA-derived appendicular skeletal muscle mass estimate (ASM) in men and women.

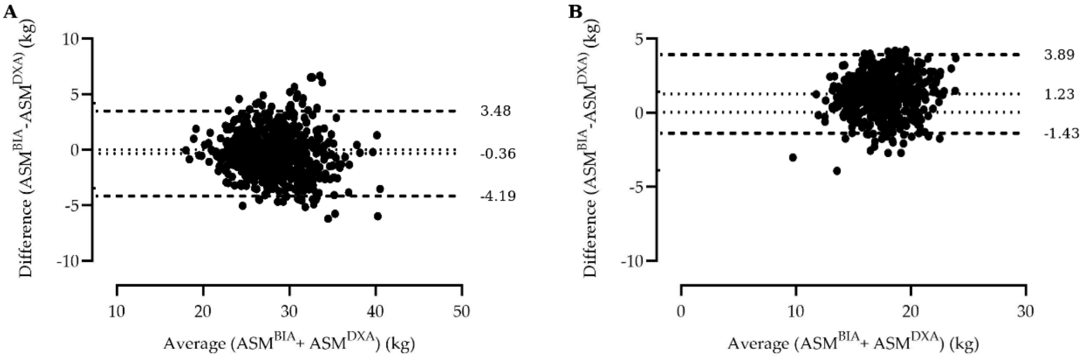

Panel A presents men, and Panel B presents women. The central dotted line represents the mean bias, calculated as MF-BIA minus DXA, and the dashed lines represent the 95% limits of agreement. Positive values indicate overestimation by MF-BIA, whereas negative values indicate underestimation by MF-BIA.

Supplementary Table S2. Exploratory age-stratified agreement between MF-BIA- and DXA-derived body composition estimates

Stratum	Age group	Outcome	Unit	n	Pearson r	Lin's CCC	Bias (SD)	95% LoA
All	18–39 years	BF%	pp	824	0.948	0.87	-3.82 (3.23)	-10.15 to 2.51
All	18–39 years	FFM	kg	824	0.979	0.951	3.01 (2.55)	-1.99 to 8.01
All	18–39 years	FM	kg	824	0.964	0.913	-2.70 (2.38)	-7.36 to 1.96
All	40–59 years	BF%	pp	325	0.958	0.867	-4.15 (2.74)	-9.52 to 1.21
All	40–59 years	FFM	kg	325	0.984	0.944	3.65 (2.27)	-0.81 to 8.11
All	40–59 years	FM	kg	325	0.973	0.925	-3.02 (2.25)	-7.44 to 1.39
All	≥60 years	BF%	pp	101	0.946	0.872	-3.57 (2.93)	-9.31 to 2.17
All	≥60 years	FFM	kg	101	0.975	0.933	3.08 (2.27)	-1.38 to 7.54
All	≥60 years	FM	kg	101	0.974	0.947	-2.32 (2.25)	-6.72 to 2.09
Men	18–39 years	BF%	pp	504	0.915	0.81	-3.58 (3.06)	-9.58 to 2.41
Men	18–39 years	FFM	kg	504	0.932	0.861	2.96 (2.70)	-2.32 to 8.24
Men	18–39 years	FM	kg	504	0.956	0.896	-2.84 (2.48)	-7.71 to 2.03
Men	40–59 years	BF%	pp	158	0.918	0.802	-3.74 (2.81)	-9.25 to 1.77
Men	40–59 years	FFM	kg	158	0.942	0.848	3.59 (2.60)	-1.50 to 8.69
Men	40–59 years	FM	kg	158	0.964	0.915	-3.16 (2.59)	-8.24 to 1.92
Men	≥60 years	BF%	pp	26	0.906	0.812	-2.82 (2.54)	-7.81 to 2.16
Men	≥60 years	FFM	kg	26	0.933	0.84	2.68 (2.25)	-1.73 to 7.09
Men	≥60 years	FM	kg	26	0.961	0.918	-2.28 (2.10)	-6.40 to 1.85
Women	18–39 years	BF%	pp	320	0.924	0.785	-4.19 (3.46)	-10.96 to 2.58
Women	18–39 years	FFM	kg	320	0.852	0.665	3.09 (2.30)	-1.42 to 7.60
Women	18–39 years	FM	kg	320	0.974	0.924	-2.47 (2.18)	-6.75 to 1.80
Women	40–59 years	BF%	pp	167	0.945	0.79	-4.54 (2.62)	-9.67 to 0.59
Women	40–59 years	FFM	kg	167	0.898	0.651	3.70 (1.92)	-0.07 to 7.47
Women	40–59 years	FM	kg	167	0.982	0.934	-2.90 (1.87)	-6.56 to 0.76
Women	≥60 years	BF%	pp	75	0.895	0.762	-3.83 (3.02)	-9.76 to 2.09
Women	≥60 years	FFM	kg	75	0.89	0.726	3.22 (2.28)	-1.25 to 7.69
Women	≥60 years	FM	kg	75	0.974	0.949	-2.33 (2.31)	-6.86 to 2.20

Note: MF-BIA: multifrequency bioelectrical impedance analysis; DXA: dual-energy X-ray absorptiometry; BF%: body fat percentage; FFM: fat-free mass; FM: fat mass; r: Pearson's correlation coefficient; CCC: concordance correlation coefficient; SD: standard deviation; LoA: limits of agreement; pp: percentage points. Age groups were examined descriptively as broad categories to avoid excessive fragmentation of the sample. Bias was calculated as MF-BIA minus DXA; positive values indicate overestimation by MF-BIA relative to DXA, whereas negative values indicate underestimation. The exploratory age-stratified analysis was performed for outcomes with complete participant-level paired data available for this sensitivity analysis.

Supplementary Table S3. Proportional-bias and Breusch-Pagan heteroscedasticity analyses

Sex	Outcome	n	Proportional-bias slope (95% CI)	Proportional-bias p	Breusch-Pagan χ^2	Breusch-Pagan p
Men	BF%	688	−0.059 (−0.086 to −0.032)	<0.001	0.19	0.659
Men	FFM	688	−0.001 (−0.029 to 0.026)	0.923	13.66	<0.001
Men	FM	688	−0.053 (−0.072 to −0.033)	<0.001	32.98	<0.001
Women	BF%	562	−0.128 (−0.155 to −0.100)	<0.001	1.57	0.211
Women	FFM	562	−0.051 (−0.094 to −0.008)	0.019	10.79	0.001
Women	FM	562	−0.075 (−0.092 to −0.059)	<0.001	24.11	<0.001

Note: BF%: body fat percentage; FFM: fat-free mass; FM: fat mass; MF-BIA: multifrequency bioelectrical impedance analysis; DXA: dual-energy X-ray absorptiometry; CI: confidence interval; χ^2: chi-square statistic. Bias was calculated as MF-BIA minus DXA. Proportional bias was assessed by regressing the between-method difference on the mean of the two methods. The Breusch–Pagan test was applied to the residuals of this model to assess whether residual variance changed across the measurement range.
